# A Multicenter Phase II RCT to Compare the Effectiveness of EMDR Versus TAU in Patients With a First-Episode Psychosis and Psychological Trauma: A Protocol Design

**DOI:** 10.3389/fpsyt.2019.01023

**Published:** 2020-02-05

**Authors:** Alicia Valiente-Gómez, Nuria Pujol, Ana Moreno-Alcázar, Joaquim Radua, Eila Monteagudo-Gimeno, Itxaso Gardoki-Souto, Bridget Hogg, Maria José Álvarez, Gemma Safont, Walter Lupo, Victor Pérez, Benedikt L. Amann, the FEP-EMDR Research Group, Rebeca Alayón

**Affiliations:** ^1^Centre Forum Research Unit, Institut de Neuropsiquiatria i Addiccions (INAD), Parc de Salut Mar, Barcelona, Spain; ^2^IMIM (Hospital del Mar Medical Research Institute), Barcelona, Spain; ^3^CIBERSAM, Madrid, Spain; ^4^Department of Psychiatry and Forensic Medicine, School of Medicine Universitat Autònoma de Barcelona, Barcelona, Spain; ^5^Institut de Neuropsiquiatria i Addiccions (INAD), Hospital del Mar, Barcelona, Spain; ^6^Institut d’Investigacions Biomèdiques August Pi i Sunyer (IDIBAPS), Barcelona, Spain; ^7^Department of Psychosis Studies, Institute of Psychiatry, Psychology & Neuroscience, King’s College London, London, United Kingdom; ^8^Department of Clinical Neuroscience, Centre for Psychiatry Research, Karolinska Institutet, Stockholm, Sweden; ^9^Mental Health Department, Vic Hospital Consortium, Barcelona, Spain; ^10^University Hospital Mutua Terrassa, Barcelona, Spain

**Keywords:** first episode psychosis, psychological trauma, post-psychotic posttraumatic stress, comorbidity, EMDR therapy, treatment as usual

## Abstract

**Background:**

Patients with a first episode psychosis (FEP) who are admitted for the first time to a psychiatric hospital frequently have experienced prior psychological trauma. Additionally, 40–80% develop posttraumatic stress symptoms, which are summarized as a post-psychotic post-traumatic syndrome (PPS). Eye Movement Desensitization and reprocessing (EMDR) therapy could be an effective psychotherapy to treat a PPS and prior psychological traumas in this population.

**Objectives:**

To assess if EMDR therapy leads to: 1) a reduction of relapses after intervention, 2) an improvement of trauma-related, psychotic and affective symptoms, 3) an improvement of overall functioning, and 4) an improvement in quality of life.

**Methods:**

This is a multicenter phase II rater-blinded randomized controlled trial in which 80 FEP patients with a history of psychological trauma will be randomly assigned to EMDR (n = 40) or to TAU (n = 40). Traumatic events will be measured by the Global Assessment of Posttraumatic Stress Questionnaire, the Cumulative Trauma Screening, the Impact of Event Scale-Revised, the Dissociative Experiences Scale, the Childhood Trauma Scale, the Holmes–Rahe Life Stress Inventory, and the Dissociative Experiences Questionnaire. Clinical symptomatology will be evaluated using the Suicide and Drug Consumption module of the International Neuropsychiatric Interview, Structured Clinical Interview for Positive and Negative Syndrome Scale, Young’s Scale for Mania Evaluation, and Beck Depression II Questionnaire. Functionality will be assessed with the Global Assessment of Functioning and the Quality of Life with the Standardized Instrument developed by the EuroQol Group. The cognitive insight and adherence to the treatment will be assessed with the Beck Cognitive Insight Scale and the Drug Attitude Inventory. All variables will be measured at baseline, post-treatment and at 12-month follow-up.

**Conclusion:**

This study will provide evidence of whether EMDR therapy is effective in reducing trauma and clinical symptoms, reducing relapses and in improving functionality and quality of life in patients with FEP and a history of trauma.

**Clinical Trial Registration:**

www.ClinicalTrials.gov, identifier: NCT03991377

## Introduction

Outcomes in patients with schizophrenia spectrum disorders and bipolar disorder have remained suboptimal (1, 2). One factor contributing to this is the high prevalence of psychological trauma and adverse life events in psychosis sufferers, nearly four times the rate in the general population (3). Evidence suggests traumatic life events are an important environmental factor implicated in an increased risk for the onset of a range of psychotic spectrum disorders, including first episode of psychosis (FEP) (4, 5) and affective psychosis in bipolar disorder (6). Additionally, psychological trauma in patients with psychosis is associated with poorer service engagement (7), worse outcomes in the form of longer stays in hospital and more frequent readmissions (8), more severe positive psychotic symptoms (9), and increased suicidal ideation (10).

The physiopathological mechanisms underlying the link between trauma and psychosis are not fully understood, but appear to be due to a complex interplay of trauma, genetic, epigenetic and environmental factors (4). Furthermore, the prevalence of posttraumatic stress disorder (PTSD) in psychosis was estimated at 12.4% in a meta-analysis of 20 studies (95% CI, 4.0–20.8%) (11). A diagnosis of PTSD in psychosis was associated with poorer social functioning and more severe psychiatric symptoms (12–14). Of particular note is that the experience of suffering a FEP in and of itself can constitute a highly stressful event which triggers symptoms compatible with PTSD: this clinical condition has been defined as post-psychotic post-traumatic syndrome (PPS) (15) and is estimated to occur in 40–80% of all FEP patients (16). A range of issues are potentially traumatic for FEP patients, from the experience of suffering psychotic symptoms, such as auditory hallucinations involving hearing voices, persecutory delusions or disorganized behavior, to the experience of being admitted to a psychiatric unit for the first time. This experience can involve involuntary hospitalizations and/or drug treatment, or other restrictive measures such as mechanical restraints, and is an important risk factor for traumatization (10), and is associated with feelings of anger, sadness, distrust or impotence. The risk of developing PPS following a FEP is increased in patients with prior childhood trauma (17). The presence of PPS is associated with an increased severity in psychotic symptoms (18) and concomitant clinical depression (19). Trauma hinders adherence to drug treatment (20), which is already particularly difficult in FEP patients in general (21). Thus, PPS has significant consequences at both prognostic and treatment levels (15).

All of this evidence suggests there is an urgent need to go beyond the treatment of psychotic symptoms and integrate trauma-focused psychotherapy into the care plan for FEP patients, especially in the high-risk period following hospitalization.

Eye movement desensitization and reprocessing therapy (EMDR) is a psychotherapy based on standardized protocols, which integrates the use of bilateral stimulation (such as side-to-side eye movements) with elements of cognitive-behavioral, interpersonal and body-centered therapies (22). EMDR has been integrated into the World Health Organization guide as a first-line psychotherapy for the treatment of PTSD in adults, children and adolescents (for a review, see (23). Despite the high prevalence of comorbid PTSD in psychosis and the strong empirical evidence supporting the efficacy of this therapy, patients affected by psychosis have been commonly excluded from clinical trials related to trauma (24). However, this tendency has begun to change and early evidence shows that positive and affective symptoms in severe psychiatric disorders, as well as comorbid trauma-related symptoms, can be ameliorated by EMDR therapy (25, 26). A randomized controlled multicenter study has been conducted by (25), with a sample of 155 patients with chronic psychosis and comorbid PTSD. The results of this study demonstrated the efficacy of EMDR in the treatment of chronic PTSD symptoms in patients with a lifetime diagnosis of psychosis, with the therapeutic benefits maintained at the 6-month follow-up and a reduction in adverse events and revictimization. Several sub-analyses of the same study have demonstrated the efficacy of EMDR therapy also in patients with greater dissociative symptoms (27), with psychotic symptoms, depression and social functioning (25), and also a cost-effectiveness study in favor of EMDR therapy when compared to prolonged exposure (28).

Therefore, the main objective of the study is to evaluate the efficacy and safety of EMDR therapy in patients with an affective or non-affective FEP in the reduction of relapses, as well as the improvement of clinical symptoms, its possible positive impact on functioning and adherence to pharmacological treatment.

## Methods

### Design and Study Setting

This multicenter phase II rater-blinded randomized controlled trial will involve the participation of four different centers in Catalonia: Hospital Parc de Salut Mar, Vic Hospital Consortium, University Hospital Mutua of Terrassa and Althaia Xarxa Assistencial Universitària of Manresa. The Hospital Parc de Salut Mar is a center of reference for mental health treatment and research, among other disciplines, and the collaboration with the rest of the institutions will ensure the recruitment of the complete sample.

Participants will be randomized to either treatment as usual (TAU) or to 20 individual 60-minute EMDR sessions over 5 months, as well as TAU. EMDR therapists participating in this trial are highly experienced, have received advanced training in EMDR protocols, and were recruited through face-to-face interviews. Assessments will be carried out at three time points: baseline (T0), post-treatment at 6 months (T1) and at 12-month follow-up (T2) (see **Table 1**). The clinical raters carrying out evaluations will be blind to the participants’ research condition; however this is not a double-blind study as it is not possible to mask from the patient whether they are undergoing EMDR therapy or not due to its use of bilateral stimulation.

**Table 1:** SPIRIT Flow diagram: Schedule of enrolment, interventions and assessments.

Timepoint**	Study Period			
	Enrolment	Allocation	Post-allocation	Close-out
*-t_1_ & t_0_*	0	*T_1_*	*T_2_*
**Enrolment:**			
Eligibility screen	X			
DSM-V criteria for substances	X			
TAP	X			
BDI-II	X		X	X
YMRS	X		X	X
PANSS	X		X	X
EGEP-5	X			
CTQ	X			
CTS	X			
IES-R	X		X	X
H-RLSI	X			
SUD	X		X	X
DES	X		X	X
SDQ-20	X		X	X
Informed consent	X			
**Allocation**		X		
**Interventions:**				
EMDR				
TAU				
**Assessments:**				
GAF	X		X	X
EQ-5D	X		X	X
BCIS	X		X	X
DAI	X		X	X

DSM-V, Diagnostic and Statistical Manual of Mental Disorders, Fifth Edition; TAP, World Accentuation Test; BDI-II, Beck Depression Inventory; YMRS, Young Mania Rating Scale; PANSS, Positive and Negative Syndrome Scale; EGEP-5, Global Post-Traumatic Stress Assessment; CTQ, Childhood Trauma Questionnaire; CTS, Cumulative Trauma Scale; IES-R, Scale of the impact of events reviewed; H-RLSI, The Holmes-Rahe Life Stress Inventory; SUD, Subjective Unit of Discomfort; DES, Scale of Dissociative Experiences; SDQ, Somatoform Dissociation Questionnaire; EMDR, Eye movement desensitization and reprocessing therapy; TAU, Treatment as usual; GAF, Global Assessment of Functioning Scale; EQ-5D, Standardized Instrument for Evaluating Quality of Life Associated with Health developed by the EuroQol group; BCIS, Beck Cognitive Insight Scale; DAI, Drug Attitude Inventory.

The study has been approved by the Ethic Committees of the IMIM, Parc de Salut Mar (2018/7940/I), Vic Hospital (AC 269), Hospital Mútua of Terrassa, and Althaia Xarxa Assistencial Universitària of Manresa. All study subjects will sign the informed consent before they can enroll in the study. Further details of the trial design can be also gathered from **Supplementary Material** (Standard Protocol Items: Recommendations for Interventional Trials [SPIRIT] Checklist). The trial has been registered at ClinicalTrials.gov, identifier: NCT03991377.

### Study Aim

The main outcome of this project is to analyze whether EMDR therapy, as an adjuvant to TAU, is effective in reducing relapses in patients with an affective or non-affective FEP and a history of comorbid psychological trauma associated with first hospital admission and/or previous stressful life events. A range of scales, highlighted below, will be used to determine changes in psychiatric symptoms (see *Instruments and Measures*).

The secondary outcome includes whether EMDR therapy, as an adjuvant to TAU, is effective in reducing psychiatric symptoms and post-traumatic stress symptoms, in improving the overall functionality and quality of life associated with health, as well as adherence to pharmacological treatment. A range of clinical scales will be used the evaluate changes from baseline (see *Instruments and Measures*).

### Hypotheses

1. Patients in the EMDR group will show fewer relapses compared to the control group after therapy (T1) and at the 12-month follow-up visit (T2).
2. Patients in the EMDR group will show fewer psychiatric and trauma-related symptoms compared to the control group after therapy (T1) and at the 12-months follow-up visit (T2).
3. Patients in the EMDR group will improve their functional capacity in comparison to the control group at evaluations after therapy (T1) and at the 12-months follow-up visit (T2).
4. Patients in the EMDR group will show a better adherence to pharmacological treatment in relation to the control group after therapy (T1) and at the 12-month follow-up visit (T2).

### Participants

The study sample will consist of 80 outpatients fulfilling criteria for a diagnosis of Schizophrenia or Schizophrenia Spectrum, Major Depressive Disorder with psychotic symptoms, or Bipolar I Disorder with psychotic symptoms according to DSM-5 criteria, who had never experienced psychotic symptoms prior to the current episode. The diagnosis of all participants will be carried out by physicians with extensive experience in the diagnosis of FEP.

Inclusion criteria are (1) age between 16 and 65 years at the time of first evaluation; (2) have been diagnosed with a first psychotic episode in the last year, with or without pharmacological treatment and/or psychiatric hospitalization; (3) presence of one or more traumatic events causing trauma related symptoms (Impact of Event Scale-Revised, IES-R >0 and Subjective Units of Distress, SUD >5); 4) ability to read and write in Spanish.

Exclusion criteria are: (1) acute suicidality; (2) presence of organic brain diseases; (3) previous trauma-focused therapy in the past 2 years.

### Randomization Procedure

After the baseline (T0) assessment (see later), we will randomly allocate participants to the EMDR or TAU group following a biased coin procedure (29). We want to note that in small clinical trials, simple randomization may lead to substantial imbalance in the relevant confounding factors between groups. The biased coin procedure attempts to balance these confounding factors but it still randomizes the patients and conceals their allocation (30). It has been included in the international guidelines for drug clinical trials (31) adopted by the European Community, Japan, United States FDA, Canada and Switzerland (32).

Specifically, a computer in a central location will allocate the patients as follows: (a) it will randomly allocate the first two patients to one group or the other with p = 0.5, (b) it will allocate the next patients as follows: (b1) if one group already includes at least two more patients than the other group, it will randomly allocate the patient allocated to the smallest group with p = 0.8, (b2) otherwise, it will first simulate that the patient is allocated to EMDR and calculate the sum of the between-group square standardized differences in age, sex and years of education, it will then simulate that the patient is allocated to TAU and recalculate the sum, and finally it will randomly allocate the patient to the group associated to the smallest sum with p = 0.8. Following this procedure, the final groups should be balanced in size and matched in age, sex and years of education. An independent researcher in a central location will control the computer program that will carry all steps of the randomization process.

### Computation of Sample Size

The study aims to assess the relative efficacy of an EMDR intervention protocol for patients with FEP versus TAU mainly in terms of stabilization and clinical improvement, reduction of anxious, depressive, somatic and/or psychotic symptoms, among others. For this reason, the main variable used is the number of clinical relapses after the intervention, with a follow-up of up to 12 months. Using previous studies as a basis (33), the sample size has been calculated based on a survival analysis with the statistical package “powerSurvEpi” for R, (http://www.r-project.org/) using an alpha = 0.005 instead of 0.05 to allow correction for multiple comparisons. The number of patients required to detect a hazard ratio = 2 in a Cox regression with a statistical power of 80% and alpha = 0.005 is n = 36 per intervention group (two groups, total n = 72). According to Chambless and Hollon (29), a sample of this size should show clinically relevant differences. Assuming a loss percentage of approximately 10–15% of the patients in the study, it would be necessary to recruit approximately 80 patients, 40 for each branch of intervention.

### Statistical Analysis

The distribution of socio-demographic, clinical (including psychological trauma), functionality, quality of life and treatment adherence characteristics among baseline groups will be analyzed using descriptive statistics. Continuous variables with a normal distribution will be analyzed with the Multivariate Variance Analysis. The change of the clinical variables of functionality and quality of life with respect to the baseline assessment at strategic points of the intervention will be analyzed by ANOVA with repeated measurements, including time factors, treatment conditions and their interaction. For those cases in which the premises of normality are not fulfilled, the Wilcoxon test will be used. The differences between groups, for the categorical and main clinical variables, will be analyzed by means of the Chi-square test. Those variables that are statistically significant can be used as covariates for a logistic or linear regression study of the factors associated to the magnitude of the effect and determine which variables are the best predictors of functioning. The effect size index (Hedges g or r de Pearson index) will be estimated in case of correlation indexes of each of the analyses performed. The statistical software used for the analyses will be the latest available version of the SPSS (v. 23).

For the main statistical analysis, we will conduct an intention-to-treat (ITT) analysis. We will impute lost to follow-up with the Last Observation Carried Forward (LOCF) method.

## Stepwise Procedures

### Intervention

#### EMDR

The patients in the EMDR arm of the study will receive a maximum of 20 individual 60-minute sessions, using the standard EMDR therapy protocol to treat past trauma-related symptoms developed by Francine Shapiro (34) and the Recent Traumatic Episode Protocol (R-TEP) protocol developed by Elan Shapiro (35).

Here is a brief description of the most recent eight-phase standardized protocol:

1. Patient history: The therapist collects information regarding the patient’s biography, including attachment history, medical history, physical health, as well as traumatic events experienced and their relationship to current symptoms, in order to develop a treatment plan.
2. Patient preparation: Following an explanation of EMDR therapy, the therapist checks the patient’s tolerance to different types of bilateral stimulation, primarily side-to-side eye movements, but where these are not tolerated, tapping of the patient’s hands or auditory tones can be used. Positive resources for emotional regulation are installed.
3. Patient assessment: A traumatic memory is chosen as a therapeutic target and the therapist assists the patient in identifying the image representing the worst part of the memory and its associated cognitions, emotions and corporal sensations. The patient then rates their current level of distress using the Subjective Units of Disturbance Scale (SUD), ranging from 0 (neutral or minimum disturbance) to 10 (maximum disturbance).
4. Memory desensitization: While the patient focuses on the traumatic image and associated negative cognition, emotions and physical sensations, 30–40 second sets of bilateral stimulation (e.g. through side-to-side eye movements) are applied, during which the patient observes without judgment their thoughts, and after each set tells the therapist what they observed. The therapist does not comment, instead applying further sets until the patient has processed the traumatic memory and their subjective distress is at 0 on the SUD scale.
5. Installing the positive cognition: The patient now focuses on the original traumatic memory and a positive cognition which is the opposite of the negative cognition originally generated by the traumatic memory, and further sets of bilateral stimulation are applied to install the new positive belief.
6. Body scan: While focusing on the original memory and positive belief, the patient is asked to scan their body and notice if there are any physical sensations. If there are any negative sensations, bilateral stimulation will be continued until they disappear. If there are positive sensations, short 10–12 second sets of bilateral stimulation will be applied to reinforce them.
7. Closure: At the end of the session, the therapist explains possible post-session effects, which can include new insights, memories or dreams, and what to do should these occur.
8. Reevaluation: In the following session, the therapist checks that the memory has been successfully processed and desensitized before selecting either a new traumatic memory, current trigger of distress, or potentially threatening future event to work on.

The Recent Traumatic Episode Protocol (R-TEP) developed by E. Shapiro and Laub (35) is used in this study for the treatment of a recent trauma. This protocol is a new comprehensive protocol which extends from the main protocol of F. Shapiro (34) for recent trauma with additional measures for containment and safety.

#### TAU

Once patients are discharged from the hospital, they will be treated by the multidisciplinary team of the Intensive Early Intervention Programme for Incipient Psychosis (PAE-TPI) as part of their usual treatment, which consists of a multidisciplinary approach that includes pharmacological treatment and psychological support, as well as visits with social workers or nursing staff. An individual care plan is drawn up depending on the patient’s individual needs, which may include follow-up psychiatric visits to evaluate clinical status and, if necessary, readjust pharmacological treatment, and psychological visits to assess and detect risk situations and prevent relapses using a non-trauma focused CBT. The TAU psychological treatment will not under any circumstances be focused on PTSD or trauma symptoms.

### Dropouts and Follow-Up

Any patient who is admitted to hospital following a clinical relapse during the 5-month intervention period will be considered to be dropout and will be excluded from the trial. In the case of relapse during follow-up, patients will be removed from the study and relapse-related factors will be collected for subsequent analysis.

## Materials and Equipment

### Instruments and Measures

Trauma-related symptoms will be evaluated using the instruments listed in **Table 2**:

1. Childhood Trauma Questionnaire (CTQ): The CTQ (36), Spanish validation (37), is a self-administered 28-item scale to measure abuse and neglect suffered in childhood on five subscales: emotional, physical or sexual abuse, and emotional or physical neglect, each subscale scored on a 5-point Likert scale. The score for each subscale classifies the severity of the abuse and neglect as: “none to minimal,” “low to moderate,” “moderate to severe” and “severe to extreme”.
2. Global Assessment of Posttraumatic Stress Questionnaire (EGEP-5): The EGEP-5 (38) is a 55-item clinician-applied scale to determine PTSD diagnosis, based on the current DSM-V criteria. This scale consists of three sections: events, symptoms and functioning.
3. The Holmes-Rahe Life Stress Inventory (39); Spanish validation (40) is used to determine which common stressful life events a patient has experienced in the last 12 months, with each life event scored according to a standardized measure of their impact (41) and a total score provided by summing all those applicable to the patient. Although individual characteristics and coping skills should be taken into account when interpreting scores, scores below 150 reflect low levels of stress, scores between 150 and 299 represent a 50% risk of a stress-related illness in the near future and scores above 300 represent an 80% risk (39).
4. Impact of Event Scale - Revised (IES-R): The IES-R (42), Spanish validation (43), is a 22-item self-report measure used to determine the level of subjective distress the subject has experienced over the past week related to a specific stressful life event, with items corresponding directly to 14 of the 17 DSM-IV symptoms of PTSD. Each item is scored on a 5-point Likert scale from 0 to 4, yielding a total score ranging from 0 to 88, with subscale scores for Intrusion, Avoidance and Hyperarousal. Higher scores represent greater subjective distress.
5. Dissociative Experiences Scale (DES): The DES (36), Spanish validation (44), is a 28-item self-report scale which measures the frequency with which an individual experiences a range of dissociative experiences, from normal to pathological. An overall mean score ranges from 0 to 100, and there are subscales for amnesia, dissociation and depersonalization. A total score of over 30 indicate high levels of dissociation.
6. Dissociative Experiences Questionnaire (SDQ-20): The SDQ-20 (45), Spanish validation (46), is a 20-item self-report scale used to measure somatoform dissociation. Each item refers to a physical symptom and is scored from 1 to 5 on a 5-point Likert scale, ranging from “1 = this applies to me not at all” to “5 = this applies to me extremely”, and the patient is then asked if there is a known physical cause. Total scores can range from 20 to 100, with higher scores denoting higher levels of somatoform dissociation.

**Table 2:** Measurements to evaluate trauma-related symptoms.

Clinical variable	Measurement interview/self-report	T0 Baseline	T1 Post-treatment 6 months	T2 Post-treatment 12 months
Childhood T.	CTQ	x		
PTSD	EGEP-5	x	x	x
Traumatic events	H-RLSI	x		
Trauma’s impact	IES-R	x	x	x
Dissociation	DES	x	x	x
Somatoform D.	SDQ-20	x	x	x

Childhood T, Childhood trauma; CTQ, Childhood Trauma Questionnaire; PTSD, post-traumatic stress disorder; EGEP-5, Global Assessment of Posttraumatic Stress Questionnaire; H-RLS,: The Holmes-Rahe Life Stress Inventory; IES-R, Impact Event Scale; DES, Scale of Dissociative Experiences; Somatoform D., Somatoform Dissociation; SDQ-20, Dissociative Experiences Questionnaire.

The diagnosis, clinical symptoms and level of functioning are to be assessed using the following instruments (see **Table 3**):

1. Suicide Module of Mini-International Neuropsychiatric Interview (MINI) (47): This is a short structured diagnostic interview, validated in Spain (48), which assesses the risk of suicide.
2. A diagnosis of substance use disorder will be made according to DSM-5 criteria; the alcohol abuse and dependence and substance abuse and dependence modules of the MINI will also be used to determine diagnosis (48).
3. Beck Depression Inventory (BDI-II): The BDI-II (49, 50), Spanish validation (51), is a 21-item clinician-administered scale designed for use with patients with a prior depression diagnosis, to quantitatively assess changes in the severity of depressive symptoms. Scores are summed and the total score indicates: 0–13 minimal depression; 14–19 mild depression; 20–28 moderate depression and 29–63 severe depression.
4. Young Mania Rating Scale (YMRS): The YMRS (52), Spanish validation (53), is an 11-item clinician-applied scale which quantifies the severity of manic and hypomanic symptoms. Four items are given more weight to compensate for a lack of cooperation in acute patients and are graded on a 0 to 8 scale (irritability, speech, thought content and disruptive/aggressive behavior), while the remaining seven items are scored from 0 to 4. The scores are interpreted as follows: ≤12 indicates remission; 13–19 indicates minimal symptoms; 20–25 indicates mild mania, 26–37 indicates moderate mania and 38–60 indicates severe mania.
5. Positive and Negative Symptom Scale (PANSS): The PANSS (54), Spanish validation (55), is an 30-item clinician-administered scale which measures positive, negative and general psychopathological symptoms on a scale of 1–7, based on the severity of the symptom. The semi-structured sci-PANSS interview (56), available in Spanish, will be used to facilitate the PANSS score.
6. Global Assessment of Functioning Scale (GAF): The Spanish validation version of the GAF, which uses the acronym EEAG (57), is a brief measure of overall psychological disturbance, providing a global score ranging from 0 to 100. The lower the score, the poorer the functional status.

**Table 3:** Measurements to evaluate diagnosis, clinical symptoms, and functionality.

Clinical variable	Measurement interview/self-report	T0 baseline	T1 Post-treatment 6 months	T2 Post-treatment 12 months
Diagnosis suicide	MINI	x		
Diagnosis S.	MINI	x		
Depression	BDI-II	x	x	x
Mania	YMRS	x	x	x
Psychotic S.	PANSS	x	x	x
Functionality	GAF	x	x	x

MINI, Mini-International Neuropsychiatric Interview; Diagnosis S, Diagnosis Substance; BDI-II, Beck Depression Inventory—II; YMRS, Young Mania Rating Scale; Psychotic S, Psychotic Symptoms; PANSS, Positive and Negative Symptom Scale; GAF, Global Assessment of Functioning Scale.

Quality of life associated with health, cognitive insight and adherence to treatment will be assessed using the following instruments (see **Table 4**):

1. Standardized Instrument for Evaluating Quality of Life Associated with Health developed by the EuroQol group (EQ-5D): EQ-5D is a standardized instrument developed by the EuroQol Group (58), available in a validated Spanish version (59), which provides a measure of health-related quality of life that can be used in a wide range of health conditions and treatments. It is comprised of five dimensions: mobility, self-care, usual activities, pain/discomfort and anxiety/depression. The total score ranges from 0 to 100, with lower scores indicating a poorer health-related quality of life.
2. Beck Cognitive Insight Scale (BCIS): The BCIS (60), Spanish validated version (61), is a 15-item self-report scale designed to measure the level of awareness of having a mental disorder and the need for treatment. The scale comprises two sub-scales: self-reflectiveness (nine items) and self-certainty (six items), and a composite score of cognitive insight is given by subtracting self-certainty items from those pertaining to self-reflectiveness.
3. Drug Attitude Inventory (DAI-10): The DAI-10 (62), Spanish validated version (63), is a 10-item scale for measuring attitude towards medication, with a total score obtained by summing the 10 items. In items 1, 3, 4, 7, and 10, if the answer is correct the score will be 2 and if it is false will be 1. In the rest of the items the correct ones score 1 and the false ones 2. The total score can oscillate between 10 and 20. There is no cut-off point but the higher the score, the more positive the perceived effect of the medication.

**Table 4:** Quality of life, cognitive insight, and adherence to treatment.

Clinical variable	Measurement interview/self-report	T0 baseline	T1 Post-treatment 6 months	T2 Post-treatment 12 months
Quality life	EQ-5D	x	x	x
Insight	BCIS	x	x	x
Drug attitude	DAI-10	x	x	x

EQ-5D, Standardized Instrument for Evaluating Quality of Life Associated with Health developed by the EuroQol group; BCIS, Beck Cognitive Insight Scale; DAI-10, Drug Attitude Inventory.

## Discussion

There is a relevant and growing interest in establishing a consensual and protocolled treatment approach in patients who have presented a FEP. For this reason, the tendency of public mental health network is to form high-risk psychosis and FEP care units (64), employing a multidisciplinary treatment approach including psychiatric, psychological, nursing and social interventions. While the main objective from a medical perspective is focused on alleviating the patients’ psychiatric symptoms, psychosocial approaches mainly aim to support patients and improve their insight and adherence through psychoeducation and disease awareness. The results for specific early interventions in early phase psychosis are more effective than treatment as usual (65), but usually do not include assessment and treatment of the presence of comorbid prior or current psychological trauma. As stated before, patients with psychosis have a high prevalence of psychological trauma during their lifetime which has an important negative impact on the course and prognosis of the disease (3). Beyond the high prevalence of psychological trauma, the diagnosis of a FEP and psychiatric admission represents *per se* a further risk factor of developing another vital traumatic event (15), in this case PPS. Taking this into account, it is important to explore new psychological approaches focusing on psychological trauma to reduce its impact in these patients. More recent psychotherapies, such as EMDR, have achieved increasing popularity and interest from clinicians due to their ability to integrate cognitive, emotional and behavioral components within the same approach. Preliminary evidence suggests that EMDR is as effective and safe as exposure therapy in patients with PTSD and chronic schizophrenia (66, 67) and other severe mental disorders (23, 26). Up to now, to the best of our knowledge, no study has investigated a trauma-focused intervention in FEP patients. The objective of this first trial of this character is to provide further evidence of a positive effect of EMDR in this population in a large RCT, in terms of relapse reduction and improvement in clinical variables. The strength of our study is the inclusion of a 1-year follow-up to test if possible improvements are maintained. With the results from a large RCT, we aim to promote trauma-oriented therapies in patients with a FEP that could be included in specific care units designed to treat this population. Although it has been shown that there is a clear association between PTSD and psychotic disorders, most mental health care programs do not offer trauma-oriented therapies for patients with a FEP so far.

## Limitations

One limitation of this study is the inclusion of both affective and non-affective FEP, as well as comorbid substance use disorders, leading to a more heterogeneous sample. These inclusion criteria were decided on to ensure the study was carried out on a representative real-world population. We suggest this limitation can be addressed by matching the samples in both arms. Additionally, the main underlying clinical variable common to all patients is the presence of comorbid psychological trauma. Another potential source of bias is the lack of control regarding pharmacological treatment. To partly overcome this limitation, a ‘pharmacological treatment’ variable will be included and, to the extent it is possible, patients in the study should not have their drug therapy altered once they are stabilized. As our unit has extensive experience in treating FEP patients and uses standardized treatment protocols, this will also help limit this as a potential source of bias.

## Ethics Statement

The study has been approved by the Ethics Committee of the IMIM, Parc de Salut Mar (2018/7940/I), by the Vic Hospital (AC 269), Hospital Mutua of Terrassa, and Althaia Xarxa Assistencial Universitària of Manresa (CEI 19/62).

## FEP-EMDR Research Group

The group is comprised of: Rebeca Alayón, Montserrat Coll, Jairo Santiago García Eslava, Ezequiel Pérez Sánchez, Carla Llimona, Cristina Macias, Anna Mané, Lorena Marín, Clara Monserrat, Miriam Morales, Ana María Rodríguez, Roberto Sánchez, Amira Trabsa, Daniel Bergé.

## Author Contributions

NP and BA had the idea for the project. NP, BA, AM-A and AV-G have contributed to the design of the study. AV-G wrote the first draft of the manuscript, with supervision from NP, AM-A and BA (primary supervisor). JR will carry out the randomization of patients and the statistical analyses. JR, NP, BA, AM-A, WL, EM-G, IG-S, BH, MA, GS and VP contributed to the revisions and modifications of the manuscript and all have approved the final version.

## Funding

This work was supported by a grant from the Plan Nacional de I+D+i and co‐funded by the Instituto de Salud Carlos III Subdirección General de Evaluación y Fomento de la Investigación with a Research Project to BA (PI18/00009). Also, we want to thank to Instituto de Salud Carlos III for the support to this project by the contract Juan Rodés of AVG (JR19/00001). The sources of funding had no influence on the design and the conducting and reporting of the trial. We further acknowledge the generous support by the Centro de Investigación Biomédica en Red de Salud Mental (CIBERSAM), Madrid, Spain.

## Conflict of Interest

BA is the Research Committee Chair of EMDR Europe and he has been invited as speaker to various national and international congresses of EMDR. BA and AM-A have been invited to belong to the international council of scholars of EMDR.

The remaining authors declare that the research was conducted in the absence of any commercial or financial relationships that could be construed as a potential conflict of interest.
