# Corrigendum: A Multicenter Phase II RCT to Compare the Effectiveness of EMDR Versus TAU in Patients With a First-Episode Psychosis and Psychological Trauma: A Protocol Design

**DOI:** 10.3389/fpsyt.2020.00283

**Published:** 2020-04-14

**Authors:** Alicia Valiente-Gómez, Nuria Pujol, Ana Moreno-Alcázar, Joaquim Radua, Eila Monteagudo-Gimeno, Itxaso Gardoki-Souto, Bridget Hogg, Maria José Álvarez, Gemma Safont, Walter Lupo, Victor Pérez, Benedikt L. Amann, the FEP-EMDR Research Group, Rebeca Alayón

**Affiliations:** ^1^ Centre Forum Research Unit, Institut de Neuropsiquiatria i Addiccions (INAD), Parc de Salut Mar, Barcelona, Spain; ^2^ IMIM (Hospital del Mar Medical Research Institute), Barcelona, Spain; ^3^ CIBERSAM, Madrid, Spain; ^4^ Department of Psychiatry and Forensic Medicine, School of Medicine, Universitat Autònoma de Barcelona, Barcelona, Spain; ^5^ Institut de Neuropsiquiatria i Addiccions (INAD), Hospital del Mar, Barcelona, Spain; ^6^ Institut d’Investigacions Biomèdiques August Pi i Sunyer (IDIBAPS), Barcelona, Spain; ^7^ Department of Psychosis Studies, Institute of Psychiatry, Psychology & Neuroscience, King’s College London, London, United Kingdom; ^8^ Department of Clinical Neuroscience, Centre for Psychiatry Research, Karolinska Institutet, Stockholm, Sweden; ^9^ Mental Health Department, Vic Hospital Consortium, Barcelona, Spain; ^10^ University Hospital Mutua Terrassa, Barcelona, Spain

**Keywords:** first episode psychosis, psychological trauma, post-psychotic posttraumatic stress, comorbidity, EMDR therapy, treatment as usual

An author’s name was incorrectly spelled as “Daniel Vergé” in the FEP-EMDR Research Group section. The correct spelling is “Daniel Bergé.” The authors apologize for this error and state that this does not change the scientific conclusions of the article in any way.

The original article has been updated.

